# Unlocking the potential for the quality of life in older adults from Kazakhstan during the COVID-19 pandemic: modeling study on gender and place of residency and associated factors

**DOI:** 10.3389/fmed.2025.1682459

**Published:** 2026-01-22

**Authors:** Assel Izekenova, Dinara Sukenova, Aigulsum Izekenova, Ardak Nurbakyt, Dejan Nikolic, Jurate Macijauskiene, Yineng Chen, Maiya Zhakupova, Maikul Kainarbayeva, Sindi Mitrovic, Natasa Radosavljevic, Saeed Alzahb, Jovana Kuzmanovic Pficer, Fei Sun

**Affiliations:** 1 Center for Social and Business Research, Kenzhegali Sagadiyev University of International Business, Almaty, Kazakhstan; 2 School of Social Work, Michigan State University, East Lansing, MI, United States; 3 Department of Public Health, Asfendiyarov Kazakh National Medical University, Almaty, Kazakhstan; 4 Department of Epidemiology with the Course of HIV, Asfendiyarov Kazakh National Medical University, Almaty, Kazakhstan; 5 Faculty of Medicine, University of Belgrade, Belgrade, Serbia; 6 Department of Physical Medicine and Rehabilitation, University Children’s Hospital, Belgrade, Serbia; 7 Department of Geriatrics, Lithuanian University of Health Sciences, Kaunas, Lithuania; 8 Department of Epidemiology and Biostatistics, College of Integrated Health Science, University at Albany, Albany, NY, United States; 9 Academician T. Sharmanov Department of Nutrition, Asfendiyarov Kazakh National Medical University, Almaty, Kazakhstan; 10 Clinic for Rehabilitation “Dr Miroslav Zotovic”, Belgrade, Serbia; 11 Department of Biomedical Sciences, State University of Novi Pazar, Novi Pazar, Serbia; 12 King Faisal Medical City, Aseer Rehabilitation Center, Abha, Saudi Arabia; 13 Department for Medical Statistics and Informatics, School of Dental Medicine, University of Belgrade, Belgrade, Serbia

**Keywords:** COVID-19, health-related factors, older adults, quality of life, sociodemographic factors

## Abstract

**Background:**

This study aimed to evaluate sociodemographic and health-related factors associated with quality of life (QoL) in older adults from Kazakhstan during the COVID-19 pandemic, as well as gender- and place-of-residence differences in these factors associated with QoL.

**Method:**

This study included 445 individuals aged 60 years and above from both urban and rural areas in Kazakhstan, between June and July 2022. Sociodemographic and health-related variables were analyzed. QoL was evaluated by the Older People’s Quality of Life (OPQoL) Scale.

**Results:**

The multivariate regression analysis indicated that the entire study population belonging to an ethnic group other than Kazakh (*p* < 0.001) was associated with lower OPQoL scores. For men, being married (*p* = 0.001) was significantly associated with the higher OPQoL scores, while belonging to an ethnic group other than Kazakh (*p* = 0.040) was associated with the lower OPQoL scores. Regarding women, the presence of COPD (*p* < 0.001) and belonging to an ethnic group other than Kazakh (*p* < 0.001) were significantly associated with the lower OPQoL scores. For those living in urban areas, better self-reported overall health (*p* = 0.005) and the absence of chronic heart failure (*p* = 0.041) were significantly associated with higher OPQoL scores. In comparison, the presence of chronic diseases (*p* < 0.001) and belonging to an ethnic group other than Kazakh (*p* < 0.001) were associated with lower OPQoL scores. In the rural area, in univariate regression analysis, only diabetes (*p* = 0.012) was significantly associated with lower OPQoL scores. General linear model analysis indicated that age, in combination with various health and sociodemographic factors, significantly affected OPQoL scores. In men, significant associations involved age with diabetes (*p* = 0.024) and marital status (*p* < 0.001), and in women, age with COPD (*p* = 0.005), chronic diseases (*p* = 0.014), and ethnic background (*p* < 0.001). Among urban residents, age was significantly associated with chronic heart failure (*p* = 0.021), chronic disease (*p* = 0.005), and ethnic background (*p* < 0.001), while among rural residents, age was significantly associated with hypertension (*p* = 0.024) and chronic diseases (*p* = 0.043).

**Conclusion:**

Our findings suggested that various sociodemographic and health-related factors influence QoL in older adults. Furthermore, this study showed gender- and place of residence-differentiated predictors of QoL. These results call for gender and place-of-residence-responsive healthcare provision and community support services.

## Background

The outbreak of the COVID-19 pandemic affected all spheres of people’s lives globally, with devastating effects on all aspects of population functioning and quality of life. Reports from 03 October 2023 pointed out that there were more than 1.4 million cases with confirmed COVID-19 infection, and 19,071 deaths in Kazakhstan (1). In the study by Marzo et al. (2), it was reported that COVID-19 adversely affected quality of life (QoL) across different age groups and professions. According to the 2021 population and housing census in Kazakhstan, 12.8% of the total population was 60 years and older (3). Previously, it was pointed out that older adults are more susceptible to COVID-19, with a higher prevalence of mortality related to COVID-19 in the age group 60 years and older compared with other population groups (4). During the COVID-19 pandemic, numerous countries have proposed measures to reduce the spread of the virus (2, 5, 6). Among them are social distancing, employment reductions, changes in everyday activities, and medical service restrictions that affect an individual’s psychological wellbeing (2).

Pandemic measures resulted in profound changes in lifestyle in older people, with the potential to affect the physical and mental health of individuals, particularly for those with disabilities, chronic diseases, and geriatric syndromes (6). In a population-based study in Finland, it was observed that during the early phase of the COVID-19 pandemic, the QoL of older people remained stable, even as negative changes in QoL-related factors were reported (7). A possible explanation for this phenomenon is that older people have a greater capacity to cope with environmental stressors than younger people (7).

In previous studies, numerous factors were reported to affect the QoL of older adults during the COVID-19 pandemic (8–10). In a cross-sectional study by Sürmeli et al. (8), it was noted that age, anxiety, fear, and education level were predictors of QoL in older people. In another study, poorer QoL among older adults during the COVID-19 pandemic was found to be associated with older age, lower education level, being single, unemployment, chronic disease, and self-reported functional constipation (9). Additionally, in a cross-sectional study, in Brazilian older adults with long COVID-19 syndrome, significant predictors of QoL were shown to be race, daily screen time, owning a home, musculoskeletal and anxiety symptoms, and working status (10). Differences between sexes and QoL were described in a cross-sectional online survey of community-dwelling older Canadians during the COVID-19 pandemic, in which ethnicity, region of residence, and social contact were associated with QoL in women (11). In contrast, social engagement was associated with QoL in both sexes (11). In the study on global AGEing and adult health, the results from community-dwelling older adults in China, India, Russia, South Africa, and Ghana demonstrated that social cohesion in men was significantly associated with higher QoL, while in women, being married was significantly associated with higher QoL except for women from Ghana (12).

In a population-based study on the elderly from Brazil, the authors reported a higher prevalence of chronic diseases among the elderly, and that type of disease is associated with the degree of impact on health-related quality of life (13). Furthermore, the literature reports that older people aged 65 years and above have a prevalence of multimorbidity of 67.5–87% globally (14). Additionally, female gender was positively associated with multimorbidity (15).

Moreover, it was noticed that COVID-19 reshaped the quality of life in cities, where the role of transport and land use, facilities and services, housing, urban nature, public space, and information and communication technologies (ICT) underwent transformation during COVID-19 in the quality of life in cities (16). Another study from Norway found that during COVID-19, some characteristics of compact cities were negatively associated with wellbeing and health outcomes, including higher neighborhood density, greater reliance on public transport, smaller dwellings, and reduced green space (17). In contrast, others were positively associated with wellbeing and health outcomes, including the presence of numerous local facilities (17). However, individuals from rural areas in Iowa reported lower perceived importance of social distancing and less concern about COVID-19 (18).

The primary aim of this study was to evaluate the sociodemographic and health-related factors associated with quality of life in older adults from Kazakhstan during the COVID-19 pandemic, as well as to examine gender and place of residence differences in these associations.

## Methods

### Study design and participants

This cross-sectional study included 445 individuals aged 60 years or older from both urban and rural areas in Kazakhstan. The inclusion criterion for age was 60 years or older, as the retirement age in Kazakhstan is 60 for women and 63 for men. A face-to-face survey was conducted between June and July 2022 by trained personnel. The initial study sample was 451, and the response rate was 98.7%.

### Sampling methodology and ethical considerations

In this study, a stratified sampling method was used to ensure the representativeness of the study population based on the percentage distribution of older adults aged 60 years and above in the general population of Almaty City and surrounding rural areas, as reported by the Bureau of National Statistics of the Republic of Kazakhstan (19). These areas encompass a broad range of socioeconomic and living conditions, with the highest proportion of older adults in Kazakhstan. To balance the urban and rural representation, 50% of participants were recruited from urban areas and 50% from rural areas. Within each stratum, participants were further stratified by gender, aiming for a target distribution of approximately 60% women and 40% men in the Almaty region (19).

To secure the privacy and protection of participants’ data, confidentiality was a key ethical consideration. Data collection was carried out by a team of trained interviewers. Before participating, all prospective participants were informed about the research objectives, the research team, and their rights, including the option to withdraw or discontinue the survey at any time without any penalty. Oral informed consent was obtained from all participants. To maintain anonymity, data without names were recorded in the dataset. For accuracy and quality control, all interviews were recorded, and responses were securely stored in a cloud-based system. Participants were assigned unique ID numbers in place of names. Access to the dataset was restricted to the core research team members and protected by password security to prevent unauthorized access.

### Inclusion and exclusion criteria

Inclusion criteria:

1. Adults aged 60 years and above
2. Residents of Almaty city and Almaty region

Exclusion criteria:

1. Individuals below 60 years of age
2. Individuals who refused to provide oral informed consent
3. Individuals with aphasia or significant hearing loss

### Testing instrument

The Older People’s Quality of Life (OPQoL) Scale was used to assess participants’ quality of life. This questionnaire is specifically designed for older populations and consists of 35 items in 8 categories: Life overall (4 items); Health (4 items); Social relationships (5 items); Independence, Control over life, and Freedom (4 items); Home and neighborhood (4 items); Psychological and emotional wellbeing (4 items); Financial circumstances (4 items); and Leisure and activities (6 items) (20). Participants are asked to indicate the extent to which they agree with each statement by choosing one of five options: “strongly disagree”; “disagree”; “neither agree nor disagree”; “agree”; and “strongly agree” (21). Each response was assigned a score from 1 to 5, and scores for negative statements were reversed so that higher scores indicate better QoL (21). The total OPQoL score ranges from 35 (indicating the worst possible QoL) to 175 (indicating the best possible QoL) (20).

### Evaluated variables

The information collected in the survey is as follows:

- Sociodemographic information includes age (categorized as 60–64; 65–69; 70–74; 75–79; and 80 years and above), gender (male and female), education level primary school (3–4 grades); incomplete secondary school (8–9 grades); secondary school (10–11 grades); specialized secondary (technical school and college); higher (bachelor and specialist); postgraduate (master, doctor, PhD, professor, candidate of science, doctor of science, and other); marital status (single, married, widowed, divorced, and other), number of children living with the participant (none; 1–3; 4–6; 7–9; 10 and more), ethnic background (Kazakh and other), and place of residence (urban and rural).
- Health-related information includes self-reported overall health (weak; below average; average; good; and very good), hypertension (no; yes), diabetes (no; yes), chronic heart failure (no; yes), cerebrovascular disease (no; yes), cardiovascular disease (no; yes), chronic obstructive pulmonary disease (COPD) (no; yes), and presence of chronic diseases (neurological except cerebrovascular diseases, respiratory except COPD, musculoskeletal, endocrinological except diabetes, gastrointestinal, hepatobiliary, renal, urological, and hematological diseases) (have; do not have).

For this study, we modified further variables into the following categories:

- Age group: 60–69 years, 70–79 years, and 80 years and above
- Education: Primary and secondary (Primary, Incomplete secondary, and Secondary), Specialized secondary, and University (Higher, Postgraduate, and other)
- Marital status: Single (Single, Widowed, Divorced, and other) and Married
- Number of children participants live with was formulated to “live with children”: no, yes (1 or more children).

### Statistical analysis

To assess the adequacy of the sample size, a power analysis was conducted assuming a small-to-moderate effect size (*f*^2^ = 0.10), a significance level of 0.05, and a statistical power of 80%. The results indicated that a minimum of 300 participants would be required to obtain reliable and generalizable findings. Therefore, the sample size in this study was sufficient to detect meaningful associations and to ensure the robustness of the statistical analyses.

We first compared sociodemographic and health-related characteristics between men and women as well as between individuals living in urban and rural places. Subsequently, we examined mean OPQoL scores in relation to sociodemographic and health-related characteristics for the entire sample, as well as separately for men and women and for those living in urban and rural places. Categorical variables were presented as frequencies (N) and percentages (%), while continuous variables were summarized as mean values (MVs) with standard deviations (SDs). The normality of continuous variables was assessed using the Kolmogorov–Smirnov test and graphical methods, including Q–Q plots, histograms, scatterplots, and residual plots. The Mann–Whitney U-test was used to assess statistical significance between two groups, and the Kruskal–Wallis test was applied for comparisons involving three or more groups.

In this study, all participants completed all items in the survey included in the analysis, resulting in 100% item-level completeness. No data were missing, and therefore no imputation or exclusion of responses was necessary. Data collection was conducted in person under supervision, ensuring that all items were answered.

Linear regression analysis was performed in every model to analyze associations between categorical variables and OPQoL. Coefficients were estimated by ordinary least squares (OLS), and 5,000 bootstrap resamples were used to generate robust 95% confidence intervals (CIs). All regression models were checked for standard assumptions, including linearity, normality, and homoscedasticity of residuals, and multicollinearity (VIF). No violations were observed.

Five separate models were run: (Model 1) the entire sample, (Model 2) men, (Model 3) women, (Model 4) those living in an urban setting, and (Model 5) those living in a rural setting. All variables that were significantly associated with OPQoL in the univariate linear regression were included in the multivariate linear regression analysis.

An adjusted General Linear Model (GLM) was applied with all factors included as fixed effects. Two-way interaction effects were systematically assessed by testing each primary factor (age, gender, and place of residence) against all other covariates. Stratified analyses were also conducted by gender and place of residence to explore subgroup-specific patterns. Effect sizes were reported using partial eta-squared (*η*^2^) interpreted according to Cohen (22) as small (*η*^2^ = 0.01), medium (*η*^2^ = 0.06), and large (*η*^2^ = 0.14) effects.

Statistical analysis was done using IBM SPSS statistical software (SPSS for Windows, version 26.0, SPSS, Chicago, IL, USA).

## Results

Table 1 presents the study group’s sociodemographic and health-related characteristics by gender. Frequencies across different levels of education significantly differed by gender (*p* = 0.008), presence of hypertension (*p* = 0.008), presence of chronic diseases (*p* = 0.001), and marital status (*p* < 0.001).

**Table 1:** The study group’s sociodemographic and health-related characteristics regarding gender.

Variables		Males *N* = 174	Females *N* = 271	p*
		*N* (%)	*N* (%)	
Age	60–69 years	106 (60.9)	163 (60.1)	0.797
	70–79 years	58 (33.3)	88 (32.5)	
	80 years and above	10 (5.7)	20 (7.4)	
Education	Primary and secondary	57 (32.8)	61 (22.5)	0.008
	Specialized secondary	61 (35.1)	85 (31.4)	
	University	56 (32.2)	125 (46.1)	
Self-reported overall health	Weak	5 (2.9)	8 (3.0)	0.431
	Below average	8 (4.6)	17 (6.3)	
	Average	71 (40.8)	130 (48.0)	
	Good	79 (45.4)	99 (36.5)	
	Very good	11 (6.3)	17 (6.3)	
Hypertension	No	112 (64.4)	140 (51.7)	0.008
	Yes	62 (35.6)	131 (48.3)	
Diabetes	No	159 (91.4)	245 (90.4)	0.729
	Yes	15 (8.6)	26 (9.6)	
Chronic heart failure	No	155 (89.1)	227 (83.8)	0.116
	Yes	19 (10.9)	44 (16.2)	
Cerebrovascular disease	No	171 (98.3)	257 (94.8)	0.065
	Yes	3 (1.7)	14 (5.2)	
Cardiovascular disease	No	137 (78.7)	211 (77.9)	0.827
	Yes	37 (21.3)	60 (22.1)	
COPD	No	166 (95.4)	263 (97.0)	0.363
	Yes	8 (4.6)	8 (3.0)	
Chronic diseases	Have	98 (56.3)	196 (72.3)	0.001
	Do not have	76 (43.7)	75 (27.7)	
Marital status	Single	36 (20.7)	117 (43.2)	<0.001
	Married	138 (79.3)	154 (56.8)	
Living with children	No	122 (70.1)	193 (71.2)	0.803
	Yes	52 (29.9)	78 (28.8)	
Ethnic background	Kazakh	124 (71.3)	185 (68.3)	0.503
	Other	50 (28.7)	86 (31.7)	
Place of residence	Urban	88 (50.6)	134 (49.4)	0.816
	Rural	86 (49.4)	137 (50.6)	

*—Chi-square test.

In Table 2, the study group’s sociodemographic and health-related characteristics regarding place of residence were presented. Frequencies in different levels of education (*p* < 0.001), different degrees of self-reported overall health (*p* < 0.001), presence of cerebrovascular diseases (*p* = 0.006), cardiovascular diseases (*p* = 0.001), COPD (*p* = 0.011), marital status (*p* < 0.001), living with children (*p* < 0.001), and ethnic background (*p* < 0.001) significantly differed between individuals from urban and rural places.

**Table 2:** The study group’s sociodemographic and health-related characteristics regarding place of residence.

Variables		Urban *N* = 222	Rural *N* = 223	p*
		*N* (%)	*N* (%)	
Age	60–69 years	130 (58.6)	139 (62.3)	0.692
	70–79 years	77 (34.7)	69 (30.9)	
	80 years and above	15 (6.8)	15 (6.7)	
Gender	Male	88 (39.6)	86 (38.6)	0.816
	Female	134 (60.4)	137 (61.4)	
Education	Primary and secondary	37 (16.7)	81 (36.3)	<0.001
	Specialized secondary	68 (30.63)	78 (35.0)	
	University	117 (52.7)	64 (28.7)	
Self-reported overall health	Weak	8 (3.6)	5 (2.2)	<0.001
	Below average	20 (9.0)	5 (2.2)	
	Average	113 (50.9)	88 (39.5)	
	Good	75 (33.8)	103 (46.2)	
	Very good	6 (2.7)	22 (9.9)	
Hypertension	No	123 (55.4)	129 (57.8)	0.603
	Yes	99 (44.6)	94 (42.2)	
Diabetes	No	198 (89.2)	206 (92.4)	0.245
	Yes	24 (10.8)	17 (7.6)	
Chronic heart failure	No	184 (82.9)	198 (88.8)	0.074
	Yes	38 (17.1)	25 (11.2)	
Cerebrovascular disease	No	208 (93.7)	220 (98.6)	0.006
	Yes	14 (6.3)	3 (1.4)	
Cardiovascular disease	No	159 (71.6)	189 (84.8)	0.001
	Yes	63 (28.4)	34 (15.2)	
COPD	No	209 (94.1)	220 (98.6)	0.011
	Yes	13 (5.9)	3 (1.4)	
Chronic diseases	Have	155 (69.8)	139 (62.3)	0.095
	Do not have	67 (30.2)	84 (37.7)	
Marital status	Single	97 (43.7)	56 (25.1)	<0.001
	Married	125 (56.3)	167 (74.9)	
Living with children	No	203 (91.4)	112 (50.2)	<0.001
	Yes	19 (8.6)	111 (49.8)	
Ethnic background	Kazakh	118 (53.2)	191 (85.6)	<0.001
	Other	104 (46.8)	32 (14.4)	

*—Chi-square test.

Mean OPQoL values were tested across sociodemographic and health-related groups for the entire study sample and for men and women separately, and are presented in Table 3. Among men, OPQoL mean values differed significantly across categories of self-reported overall health (*χ*^2^ = 14.73; df = 4; *p* = 0.005), with the highest mean in the “very good” category and the lowest in the “below average” category. Significantly higher OPQoL mean values were observed among men without chronic diseases (*U* = 3071.50; *z* = −1.98; *p* = 0.048), married men (*U* = 3463.50; *z* = 3.64; *p* < 0.001), and those of Kazakh ethnic background (*U* = 2256.50; *z* = −2.81; *p* = 0.005).

**Table 3:** Mean values of OPQoL score for tested variables (total score and regarding gender).

Variables		OPQoL males		OPQoL females		OPQoL total	
		MV ± SD	*p*	MV ± SD	*p*	MV ± SD	*p*
Age	60–69 years	128.93 ± 11.43	0.056*	128.77 ± 11.87	0.429*	128.83 ± 11.68	0.229*
	70–79 years	124.86 ± 14.43		127.02 ± 12.37		126.16 ± 13.22	
	80 years and above	136.90 ± 16.38		125.05 ± 13.20		129.00 ± 15.16	
Gender	Male	–	–	–	–	128.03 ± 13.05	0.872**
	Female	–		–		127.93 ± 12.14	
Education	Primary and secondary	128.09 ± 13.22	0.577*	129.77 ± 13.58	0.328*	128.96 ± 13.38	0.323*
	Specialized secondary	126.67 ± 13.24		126.94 ± 11.81		126.83 ± 12.38	
	University	129.46 ± 12.74		127.70 ± 11.61		128.24 ± 11.96	
Self-reported overall health	Weak	119.00 ± 14.49	0.005*	127.25 ± 15.72	<0.001*	124.08 ± 15.22	<0.001*
	Below average	113.38 ± 9.23		116.29 ± 11.21		115.36 ± 10.51	
	Average	128.04 ± 13.74		126.70 ± 11.80		127.17 ± 12.50	
	Good	129.38 ± 12.01		130.41 ± 11.52		129.96 ± 11.72	
	Very good	133.09 ± 9.98		134.76 ± 8.62		134.11 ± 9.04	
Hypertension	No	128.88 ± 13.13	0.317**	129.74 ± 12.68	0.004**	129.36 ± 12.87	0.005**
	Yes	126.50 ± 12.86		125.98 ± 11.26		126.15 ± 11.77	
Diabetes	No	127.65 ± 12.59	0.154**	128.18 ± 12.10	0.272**	127.98 ± 12.28	0.946**
	Yes	132.07 ± 17.17		125.50 ± 12.52		127.90 ± 14.54	
Chronic heart failure	No	128.74 ± 12.78	0.060**	129.09 ± 11.35	0.002**	128.95 ± 11.94	<0.001**
	Yes	122.32 ± 14.14		121.91 ± 14.29		122.03 ± 14.13	
Cerebrovascular disease	No	127.94 ± 13.10	0.418**	128.12 ± 12.25	0.141**	128.05 ± 12.58	0.331**
	Yes	133.33 ± 10.26		124.29 ± 9.46		125.88 ± 9.92	
Cardiovascular disease	No	129.20 ± 12.51	0.054**	129.09 ± 11.68	0.006**	129.14 ± 12.00	0.001**
	Yes	123.70 ± 14.24		123.82 ± 12.91		123.77 ± 13.36	
COPD	No	128.07 ± 12.90	0.431**	127.67 ± 12.08	0.065**	127.83 ± 12.39	0.453**
	Yes	127.25 ± 16.88		136.38 ± 11.70		131.81 ± 14.80	
Chronic diseases	Have	126.24 ± 14.19	0.048**	126.36 ± 12.47	<0.001**	126.32 ± 13.04	<0.001**
	Do not have	130.34 ± 11.07		132.03 ± 10.24		131.18 ± 10.66	
Marital status	Single	120.42 ± 12.78	<0.001**	127.13 ± 13.06	0.354**	125.55 ± 13.26	0.006**
	Married	130.02 ± 12.41		128.53 ± 11.41		129.24 ± 11.89	
Have children	No	126.98 ± 13.81	0.083**	127.36 ± 13.07	0.328**	127.21 ± 13.34	0.062**
	Yes	130.52 ± 10.78		129.33 ± 9.38		129.81 ± 9.94	
Ethnic background	Kazakh	129.86 ± 12.77	0.005**	130.65 ± 11.80	<0.001**	130.34 ± 12.18	<0.001**
	Other	123.50 ± 12.74		122.06 ± 10.76		122.59 ± 11.50	
Place of residence	Urban	126.50 ± 14.53	0.131**	126.41 ± 12.90	0.014**	126.45 ± 13.54	0.004**
	Rural	129.60 ± 11.21		129.41 ± 11.20		129.48 ± 11.18	

*—Kruskal–Wallis test; **—Mann–Whitney U-test.

Similarly, among women, OPQoL mean values differed significantly across categories of self-reported overall health (*χ*^2^ = 30.35; df = 4; *p* < 0.001), with the highest mean in the “very good” category and the lowest in the “below average” category. Higher OPQoL mean values were noted among women without hypertension (*U* = 11002.50; *z* = 2.84; *p* = 0.004), chronic heart failure (*U* = 6502.50; *z* = 3.17; *p* = 0.002), cardiovascular disease (*U* = 7788.00; *z* = 2.72; *p* = 0.006), and other chronic diseases (*U* = 5019.00; *z* = −4.04; *p* < 0.001), as well as among those of Kazakh ethnic background (*U* = 4205.00; *z* = −6.25; *p* < 0.001) and those living in rural areas (*U* = 10767.50; *z* = 2.46; *p* = 0.014).

For the entire study sample, OPQoL mean values also varied significantly across categories of self-reported overall health (*χ*^2^ = 42.26; df = 4; *p* < 0.001), with the highest mean in the “very good” category and the lowest in the “below average” category. Significantly higher OPQoL mean values were observed among participants without hypertension (*U* = 28119.00; *z* = 2.83; *p* = 0.005), chronic heart failure (*U* = 15486.50; *z* = 3.65; *p* < 0.001), cardiovascular disease (*U* = 20654.00; *z* = 3.37; *p* = 0.001), and other chronic diseases (*U* = 16698.00; *z* = −4.28; *p* < 0.001). Higher mean OPQoL values were also noted among married older adults (*U* = 25843.00; *z* = 2.72; *p* = 0.006), those of Kazakh ethnic origin (*U* = 12676.00; *z* = −6.67; *p* < 0.001), and individuals living in rural areas (*U* = 28612.00; *z* = 2.85; *p* = 0.004).

Mean OPQoL values were tested across sociodemographic and health-related groups for individuals residing in urban and rural regions and are presented in Table 4. Among individuals residing in urban regions, OPQoL mean values differed significantly across categories of self-reported overall health (*χ*^2^ = 44.51; df = 4; *p* < 0.001). Higher OPQoL mean values were noted among older adults without hypertension (*U* = 7098.50; *z* = 2.12; *p* = 0.034), chronic heart failure (*U* = 5089.50; *z* = 4.42; *p* < 0.001), cardiovascular disease (*U* = 6269.50; *z* = 2.92; *p* = 0.003), and other chronic diseases (*U* = 2828.50; *z* = −5.38; *p* < 0.001), as well as among those of Kazakh ethnic background (*U* = 3153.00; *z* = −6.25; *p* < 0.001).

**Table 4:** Mean values of OPQoL score for tested variables regarding place of residence.

Variables		OPQoL urban		OPQoL rural	
		MV±SD	*p*	MV±SD	*p*
Age	60–69 years	127.98 ± 13.41	0.169*	129.63 ± 9.77	0.651*
	70–79 years	124.06 ± 13.50		128.51 ± 12.60	
	80 years and above	125.40 ± 13.90		132.60 ± 15.97	
Gender	Male	126.50 ± 14.53	0.868**	129.60 ± 11.21	0.859**
	Female	126.41 ± 12.90		129.41 ± 11.20	
Education	Primary and secondary	125.35 ± 12.96	0.229*	130.60 ± 13.32	0.526*
	Specialized secondary	124.19 ± 14.17		129.13 ± 10.13	
	University	128.10 ± 13.23		128.50 ± 9.32	
Self-reported overall health	Weak	122.00 ± 7.13	<0.001*	127.40 ± 24.15	0.641*
	Below average	112.55 ± 9.80		126.60 ± 3.05	
	Average	124.83 ± 12.96		130.18 ± 11.27	
	Good	131.72 ± 12.25		128.67 ± 11.20	
	Very good	143.17 ± 7.99		131.64 ± 7.74	
Hypertension	No	128.38 ± 13.55	0.034**	130.29 ± 12.16	0.056**
	Yes	124.04 ± 13.20		128.37 ± 9.62	
Diabetes	No	126.97 ± 13.44	0.108**	128.94 ± 11.01	0.007**
	Yes	122.08 ± 13.84		136.12 ± 11.44	
Chronic heart failure	No	128.27 ± 13.07	<0.001**	129.58 ± 10.77	0.594**
	Yes	117.61 ± 12.36		128.76 ± 14.22	
Cerebrovascular disease	No	126.60 ± 13.76	0.439**	129.43 ± 11.22	0.330**
	Yes	124.21 ± 9.81		133.67 ± 7.23	
Cardiovascular disease	No	128.18 ± 13.40	0.003**	129.95 ± 10.65	0.433**
	Yes	122.08 ± 12.98		126.91 ± 13.67	
COPD	No	126.25 ± 13.56	0.479**	129.32 ± 11.00	0.291**
	Yes	129.62 ± 13.33		141.33 ± 20.21	
Chronic diseases	Have	123.26 ± 12.78	<0.001**	129.73 ± 12.52	0.980**
	Do not have	133.82 ± 12.39		129.07 ± 8.56	
Marital status	Single	124.79 ± 13.69	0.167**	126.86 ± 12.49	0.080**
	Married	127.73 ± 13.33		130.37 ± 10.60	
Have children	No	126.08 ± 13.78	0.132**	129.26 ± 12.32	0.754**
	Yes	130.37 ± 10.13		129.71 ± 9.95	
Ethnic background	Kazakh	131.52 ± 14.11	<0.001**	129.61 ± 10.80	0.488**
	Other	120.69 ± 10.21		128.75 ± 13.37	

*—Kruskal–Wallis test; **—Mann–Whitney U-test.

Among individuals residing in rural regions, significantly higher OPQoL mean values were observed among older adults with diabetes (*U* = 1068.00; *z* = −2.68; *p* = 0.007).

The results of the univariate and multivariate linear regression analyses of sociodemographic and health-related factors associated with OPQoL scores for the entire sample are presented in Table 5. In the univariate linear regression analysis, better self-reported overall health (*p* < 0.001), absence of hypertension (*p* = 0.007), absence of chronic heart failure (*p* < 0.001), absence of cardiovascular diseases (*p* = 0.001), being married (*p* = 0.003), having children (*p* = 0.022), and rural place of residence (*p* = 0.010) were all significantly associated with the higher OPQoL scores, while presence of chronic diseases (*p* < 0.001) and belonging to the ethnic group other than Kazakh (*p* < 0.001) were associated with the lower OPQoL scores. In the multivariate linear regression analysis, belonging to an ethnic group other than Kazakh (*p* < 0.001) was significantly associated with lower OPQoL scores. In the overall multivariate regression model, we accounted for 14% of the variance in OPQoL (*R*^2^ = 0.141, *F* = 7.931, *p* < 0.001).

**Table 5:** Sociodemographic and health-related factors associated with OPQoL in the entire sample.

Model 1 (OPQoL entire sample)	Univariate linear regression			Multivariate linear regression		
Variable	*B*	95% CI	*p*	*B*	95% CI	*p*
Age	−1.181	−3.302 to 0.813	0.247	–	–	–
Gender	−0.108	−2.548 to 2.315	0.938	–	–	–
Education	−0.231	−1.767 to 1.276	0.759	–	–	–
Self-reported overall health	3.818	2.346–5.248	<0.001	1.627	−0.277 to 3.466	0.088
Hypertension	3.211	0.959–5.571	0.007	0.013	−3.445 to 3.308	0.995
Diabetes	0.073	−4.506 to 4.583	0.975	–	–	–
Chronic heart failure	6.916	3.144–10.665	<0.001	2.503	−1.648 to 6.692	0.247
Cerebrovascular diseases	2.169	−2.796 to 7.036	0.386	–	–	–
Cardiovascular diseases	5.365	2.408–8.392	0.001	1.820	−1.723 to 5.491	0.323
COPD	−3.987	−11.614 to 3.276	0.279	–	–	–
Chronic diseases	−4.859	−7.122 to −2.638	<0.001	−1.920	−5.334 to 1.387	0.272
Marital status	3.687	1.220–6.189	0.003	2.468	−0.010 to 5.104	0.061
Having children	2.598	0.362–4.787	0.022	−0.254	−2.983 to 2.450	0.854
Ethnic background	−7.748	−10.057 to −5.377	<0.001	−6.363	−9.057 to −3.674	<0.001
Place of residence	3.038	0.788–5.314	0.010	−0.503	−3.394 to 2.340	0.727

The results of the univariate and multivariate linear regression investigations of sociodemographic and health-related factors associated with OPQoL scores in men and women are presented in Table 6. In the univariate linear regression analysis, better self-reported overall health (*p* = 0.001), absence of cardiovascular diseases (*p* = 0.030), and being married (*p* < 0.001) were significantly associated with the higher OPQoL scores, while the presence of chronic diseases (*p* = 0.036) and belonging to an ethnic group other than Kazakh (*p* = 0.003) were associated with the lower OPQoL scores. In the multivariate linear regression analysis, being married (*p* = 0.001) was significantly associated with the higher OPQoL scores, while belonging to an ethnic group other than Kazakh (*p* = 0.040) was associated with the lower OPQoL scores. In the multivariate regression model from the men subgroup, we verified 15% of the variance for OPQoL (*R*^2^ = 0.151, *F* = 5.974, *p* < 0.001).

**Table 6:** Sociodemographic and health-related factors associated with OPQoL in men and women.

Variable	Univariate linear regression			Multivariate linear regression		
	*B*	95% CI	*p*	*B*	95% CI	*p*
Model 2 (OPQoL male)						
Age	−0.106	−3.976 to 3.461	0.955	–	–	–
Education	0.682	−1.692 to 3.193	0.579	–	–	–
Self-reported overall health	3.816	1.460–6.072	0.001	1.491	−1.602 to 4.378	0.319
Hypertension	2.384	−1.591 to 6.405	0.256	–	–	–
Diabetes	−4.413	−13.042 to 4.844	0.333	–	–	–
Chronic heart failure	6.420	−0.205 to 13.220	0.055	–	–	–
Cerebrovascular diseases	−5.392	−14.665 to 6.642	0.312	–	–	–
Cardiovascular diseases	5.502	0.596–10.611	0.030	3.367	−2.290 to 9.387	0.255
COPD	0.822	−12.678 to 11.439	0.889	–	–	–
Chronic diseases	−4.097	−7.957 to −0.403	0.036	−0.512	−4.978 to 3.930	0.826
Marital status	9.605	4.959–14.265	<0.001	8.035	3.438–12.730	0.001
Having children	3.544	−0.317 to 7.375	0.074	–	–	–
Ethnic background	−6.363	−10.639 to −2.200	0.003	−4.322	−8.438 to −0.345	0.040
Place of residence	3.105	−0.749 to 6.976	0.118	–	–	–
Model 3 (OPQoL female)						
Age	−1.809	−4.080 to 0.468	0.120	–	–	–
Education	−0.827	−2.671 to 1.123	0.396	–	–	–
Self-reported overall health	3.838	1.965–5.640	<0.001	1.109	−1.171 to 3.329	0.342
Hypertension	3.758	0.862–6.650	0.012	0.636	−3.504 to 4.776	0.756
Diabetes	2.684	−2.264 to 7.887	0.296	–	–	–
Chronic heart failure	7.183	2.661–11.835	0.003	4.310	−0.857 to 9.661	0.106
Cerebrovascular diseases	3.839	−1.496 to 8.827	0.136	–	–	–
Cardiovascular diseases	5.278	1.597–9.061	0.004	1.084	−2.852 to 4.997	0.589
COPD	−8.706	−17.140 to −0.254	0.022	−13.840	−20.088 to −7.258	<0.001
Chronic diseases	−5.670	−8.517 to −2.763	<0.001	−3.105	−7.070 to 0.807	0.133
Marital status	1.404	−1.549 to 4.295	0.350	–	–	–
Having children	1.976	−0.872 to 4.778	0.162	–	–	–
Ethnic background	−8.596	−11.473 to −5.801	<0.001	−7.210	−10.380 to −3.964	<0.001
Place of residence	2.998	0.189–5.869	0.040	0.704	−2.541 to 3.954	0.677

In the univariate linear regression analysis, better self-reported overall health (*p* < 0.001), absence of hypertension (*p* = 0.012), absence of chronic heart failure (*p* = 0.003), and absence of cardiovascular diseases (*p* = 0.004), and rural residence (*p* = 0.040) were significantly associated with the higher OPQoL scores, while COPD (*p* = 0.022), presence of chronic diseases (*p* < 0.001), and belonging to the ethnic group other that Kazakh (*p* < 0.001) were associated with the lower OPQoL scores. In the multivariate linear regression analysis, the presence of COPD (*p* < 0.001) and belonging to an ethnic group other than Kazakh (*p* < 0.001) were significantly associated with the lower OPQoL scores. In the multivariate regression model from the women subgroup, we verified 20% of the variance for OPQoL (*R*^2^ = 0.201, *F* = 8.243, *p* < 0.001).

The results of the univariate and multivariate linear regressions analyzing sociodemographic and health-related factors associated with OPQoL scores for urban and rural residents are presented in Table 7. In the univariate linear regression analysis for urban residents, better self-reported overall health (*p* < 0.001), absence of hypertension (*p* = 0.017), absence of chronic heart failure (*p* < 0.001), and absence of cardiovascular diseases (*p* = 0.002) were significantly associated with the higher OPQoL scores, while the presence of chronic diseases (*p* < 0.001) and belonging to the ethnic group other that Kazakh (*p* < 0.001) were associated with the lower OPQoL scores. In the multivariate linear regression model for urban residents, better self-reported overall health (*p* = 0.005) and absence of chronic heart failure (*p* = 0.041) were significantly associated with the higher OPQoL scores. In comparison, the presence of chronic diseases (*p* < 0.001) and belonging to an ethnic group other than Kazakh (*p* < 0.001) were associated with the lower OPQoL scores. In the multivariate regression model from the urban subgroup, we verified 31% of the variance for OPQoL (*R*^2^ = 0.309, *F* = 16.009, *p* < 0.001).

**Table 7:** Sociodemographic and health-related factors associated with OPQoL in urban and rural residences.

Variable	Univariate linear regression			Multivariate linear regression		
	*B*	95% CI	*p*	*B*	95% CI	*p*
Model 4 (OPQoL urban)						
Age	−2.513	−5.395 to 0.329	0.083	–	–	–
Gender	−0.090	−3.906 to 3.641	0.962	–	–	–
Education	1.872	−0.358 to 4.164	0.104	–	–	–
Self-reported overall health	6.787	4.883–8.907	<0.001	3.545	1.129–5.983	0.005
Hypertension	4.342	0.774–7.761	0.017	−2.304	−6.213 to 1.362	0.233
Diabetes	4.891	−1.146 to 10.799	0.106	–	–	–
Chronic heart failure	10.666	6.285–15.102	<0.001	5.197	0.161–10.220	0.041
Cerebrovascular diseases	2.382	−3.136 to 7.822	0.375	–	–	–
Cardiovascular diseases	6.097	2.333–9.971	0.002	−1.780	−5.888 to 2.569	0.404
COPD	−3.367	−10.843 to 4.054	0.354	–	–	–
Chronic diseases	−10.563	−14.102 to −6.939	<0.001	−7.519	−11.797 to −3.388	<0.001
Marital status	2.934	−0.709 to 6.631	0.115	–	–	–
Having children	4.290	−0.633 to 9.154	0.081	–	–	–
Ethnic background	−10.825	−14.023 to −7.540	<0.001	−7.909	−11.131 to −4.868	<0.001
Model 5 (OPQoL rural)						
Age	0.306	−2.616 to 3.078	0.833	–	–	–
Gender	−0.196	−3.244 to 2.792	0.898	–	–	–
Education	−1.070	−2.958 to 0.777	0.254	–	–	–
Self-reported overall health	0.334	−2.008 to 2.452	0.773	–	–	–
Hypertension	1.922	−0.965 to 4.818	0.196	–	–	–
Diabetes	−7.181	−12.914 to −1.557	0.012	–	–	–
Chronic heart failure	0.816	−4.673 to 6.914	0.785	–	–	–
Cerebrovascular diseases	−4.239	−12.973 to 1.153	0.247	–	–	–
Cardiovascular diseases	3.035	−1.597 to 8.114	0.222	–	–	–
COPD	−12.011	−34.222 to 6.778	0.244	–	–	–
Chronic diseases	0.662	−2.005 to 3.466	0.627	–	–	–
Marital status	3.508	−0.185 to 7.154	0.064	–	–	–
Having children	0.453	−2.530 to 3.440	0.767	–	–	–
Ethnic background	−0.857	−5.741 to 4.135	0.726	–	–	–

In the univariate linear regression analysis for rural residents, diabetes (*p* = 0.012) was significantly associated with the lower OPQoL scores.

Adjusted GLM analyses examining the interaction effects of age, gender, and place of residence with sociodemographic and health-related variables on OPQoL scores are presented in Table 8. This general linear model analysis revealed that age, in interaction with self-reported overall health (*p* = 0.032), COPD (*p* = 0.025), chronic diseases (*p* = 0.042), marital status (*p* = 0.013), and ethnic background (*p* < 0.001), had a significant impact on quality of life, as measured by the OPQoL score. In the age interaction model, the age in interaction with self-reported overall health (*η*^2^ = 0.056) had the largest effect, followed by the interaction with ethnic background (*η*^2^ = 0.049), marital status (η^2^ = 0.027), COPD (*η*^2^ = 0.024), and chronic diseases (*η*^2^ = 0.021).

**Table 8:** Adjusted GLM analyses examining the interaction effects of age, gender, and residence with sociodemographic and health-related variables on OPQoL scores.

Variables	OPQoL								
	Age*			Gender*			Place of residence*		
	*F*	*p*	*η* ^2^	*F*	*p*	*η* ^2^	*F*	*p*	*η* ^2^
*R* ^2^	0.266			0.245			0.291		
Adjusted *R*^2^	0.160			0.172			0.223		
Age	–	–		2.447	0.046	0.024	1.291	0.273	0.013
Gender	1.915	0.127	0.015	–	–		0.928	0.396	0.005
Education	0.681	0.665	0.010	0.431	0.786	0.004	0.576	0.680	0.006
Self-reported overall health	1.908	0.032	0.056	1.910	0.057	0.036	2.349	0.018	0.044
Hypertension	1.614	0.186	0.012	0.791	0.454	0.004	1.869	0.156	0.009
Diabetes	1.276	0.282	0.010	2.703	0.068	0.013	2.763	0.064	0.013
Chronic heart failure	1.980	0.116	0.015	2.167	0.116	0.011	5.133	0.006	0.025
Cerebrovascular disease	1.581	0.194	0.012	1.542	0.215	0.008	1.326	0.267	0.007
Cardiovascular diseases	0.958	0.412	0.007	0.420	0.657	0.002	1.078	0.341	0.005
COPD	3.158	0.025	0.024	5.597	0.004	0.027	3.832	0.022	0.019
Chronic diseases	2.762	0.042	0.021	1.962	0.142	0.010	7.042	0.001	0.034
Marital status	3.613	0.013	0.027	7.016	0.001	0.033	3.225	0.041	0.016
Living with children	0.094	0.963	0.001	0.106	0.899	0.001	1.658	0.192	0.008
Ethnic background	6.680	<0.001	0.049	10.027	<0.001	0.047	9.624	<0.001	0.045
Place of residence	0.206	0.892	0.002	0.005	0.995	<0.001	–	–	–

*η*^2^ = partial eta-squared (effect size), *—two-way interactions between age, gender, or place of residence and all covariates (fixed effects).

Furthermore, gender in interaction with age (*p* = 0.046), COPD (*p* = 0.004), marital status (*p* = 0.001), and ethnic background (*p* < 0.001) significantly affected OPQoL scores. In the gender interaction model, the gender in interaction with ethnic background (*η*^2^ = 0.047) had the largest effect, followed by the interaction with marital status (*η*^2^ = 0.033), COPD (*η*^2^ = 0.027), and age (*η*^2^ = 0.024).

Additionally, place of residence in interaction with self-reported overall health (*p* = 0.018), chronic heart failure (*p* = 0.006), COPD (*p* = 0.022), chronic diseases (*p* = 0.001), marital status (*p* = 0.041), and ethnic background (*p* < 0.001) also had a significant impact on OPQoL scores. In the place of residence interaction model, the place of residence in interaction with ethnic background (*η*^2^ = 0.045) had the largest effect, followed by the interaction with self-reported overall health (*η*^2^ = 0.044), chronic diseases (*η*^2^ = 0.034), chronic heart failure (*η*^2^ = 0.025), COPD (*η*^2^ = 0.019), and marital status (*η*^2^ = 0.016).

Tests of between-subjects effects for OPQoL in relation to age across different genders and places of residence are presented in Table 9. The general linear model analysis demonstrated that, in men, age in interaction with diabetes (*p* = 0.024) and age in interaction with marital status (*p* < 0.001) had a significant impact on quality of life measured by the OPQoL score. In women, significant interactions were observed between age and COPD (*p* = 0.005), chronic diseases (*p* = 0.014), and ethnic background (*p* < 0.001). In the male gender interaction model, the age in interaction with marital status (*η*^2^ = 0.123) had the largest effect, followed by the interaction with diabetes (*η*^2^ = 0.057), while in the female gender interaction model, the age in interaction with ethnic background (*η*^2^ = 0.111) had the largest effect, followed by the interaction with COPD (*η*^2^ = 0.056) and chronic diseases (*η*^2^ = 0.047).

**Table 9:** Tests of between-subjects effects for OPQoL regarding age between different genders and place of residency.

Variables	OPQoL							
	Gender				Place of residency			
	Type	*F*	*p*	*η* ^2^	Type	*F*	*p*	*η* ^2^
Age*gender	Male	–	–	–	Urban	1.797	0.169	0.021
	Female	–	–	–	Rural	0.383	0.766	0.007
Age*education	Male	0.821	0.514	0.025	Urban	0.793	0.556	0.023
	Female	1.562	0.159	0.041	Rural	0.459	0.807	0.013
Age*self-reported overall health	Male	0.930	0.494	0.055	Urban	1.694	0.094	0.082
	Female	1.393	0.177	0.066	Rural	0.619	0.797	0.034
Age*hypertension	Male	1.211	0.301	0.019	Urban	0.482	0.618	0.006
	Female	2.277	0.081	0.030	Rural	3.232	0.024	0.053
Age*diabetes	Male	3.836	0.024	0.057	Urban	0.818	0.443	0.009
	Female	2.179	0.091	0.029	Rural	2.136	0.097	0.036
Age*chronic heart failure	Male	2.106	0.126	0.032	Urban	3.940	0.021	0.044
	Female	1.661	0.176	0.022	Rural	0.520	0.669	0.009
Age*cerebrovascular disease	Male	1.970	0.144	0.030	Urban	0.287	0.751	0.003
	Female	0.532	0.661	0.007	Rural	0.976	0.325	0.006
Age*cardiovascular disease	Male	0.479	0.620	0.007	Urban	0.346	0.708	0.004
	Female	1.416	0.239	0.019	Rural	2.246	0.109	0.025
Age*COPD	Male	0.675	0.511	0.010	Urban	1.449	0.230	0.025
	Female	4.350	0.005	0.056	Rural	0.060	0.942	0.001
Age*chronic diseases	Male	1.466	0.235	0.022	Urban	5.402	0.005	0.059
	Female	3.620	0.014	0.047	Rural	2.780	0.043	0.046
Age*marital status	Male	8.950	<0.001	0.123	Urban	2.241	0.109	0.026
	Female	0.571	0.634	0.008	Rural	2.240	0.110	0.025
Age*living with children	Male	1.816	0.167	0.028	Urban	0.976	0.379	0.011
	Female	0.611	0.608	0.008	Rural	0.222	0.801	0.003
Age*ethnic background	Male	0.562	0.571	0.009	Urban	9.203	<0.001	0.097
	Female	9.086	<0.001	0.111	Rural	1.110	0.347	0.019
Age*place of residence	Male	1.837	0.164	0.028	Urban	–	–	–
	Female	0.916	0.434	0.012	Rural	–	–	–

*η*^2^ = partial eta-squared (effect size), *—reported values refer to two-way interactions between age and all covariates (fixed effects).

Among participants living in urban areas, age in interaction with chronic heart failure (*p* = 0.021), chronic diseases (*p* = 0.005), and ethnic background (*p* < 0.001) had a significant impact on OPQoL scores. In contrast, among those living in rural areas, age in interaction with hypertension (*p* = 0.024) was significant, as well as age with chronic diseases (*p* = 0.043). In the urban interaction model, the age in interaction with ethnic background (*η*^2^ = 0.097) had the largest effect, followed by the interaction with chronic diseases (*η*^2^ = 0.059) and chronic heart failure (*η*^2^ = 0.044), while in the rural interaction model, the age in interaction with hypertension (*η*^2^ = 0.053) had the largest effect, followed by the interaction with chronic diseases (*η*^2^ = 0.046).

The *R*^2^ values for the men and women samples were 0.444 and 0.348, respectively. The *R*^2^ values for the urban and rural population samples were 0.451 and 0.308, respectively. The adjusted *R*^2^ values for the men and women samples were 0.248 and 0.192, respectively. The adjusted *R*^2^ values for the urban and rural population samples were 0.290 and 0.117, respectively.

## Discussion

Before COVID, a 2014 study from Kazakhstan reported that circulatory and respiratory diseases were more common among urban than rural residents (23). Our findings during the COVID pandemic were similar to those of a previous report (23), which demonstrated that cardiovascular diseases and COPD were more frequent in urban than in rural areas.

According to the report analyzing results from the Regional Well-Being Survey of Kazakhstan, conducted by the Economic Research Institute between August and November 2022, it was stated that those living in Almaty city were more satisfied with their lives (89%) then those living in the Almaty region (78%) (24). Additionally, 57% of respondents from Almaty city were satisfied with the quality of health care in their region, compared to 41% of respondents from Almaty region (24). Moreover, according to the study by Panzabekova and Digel (25), life expectancy in the City of Almaty increased from 68.41 years in 2001 to 75.54 years in 2018, while in the Almaty region, it increased from 67.42 years in 2001 to 73.44 years in 2018. Additionally, the authors stated that, aside from economic factors, which have the most decisive influence on life expectancy in Kazakhstan, other factors related to life expectancy and quality of life begin to gain prominence after reaching a certain level of socio-economic development (25). These data indicate the need to evaluate other possible factors that may influence the quality of life of people from Kazakhstan.

During the tested time period in 2022, our results demonstrated from the univariate linear regression that better self-reported overall health, absence of hypertension, absence of chronic heart failure, absence of cardiovascular diseases, being married, having children, and rural place of residence were all significantly associated with better quality of life, while the presence of chronic diseases and belonging to ethnic group other that Kazakh were significantly associated with decreased quality of life. However, in the multivariate linear regression model, belonging to an ethnic group other than Kazakh was associated with decreased quality of life.

In older adults aged 75–99 years, specific self-reported health parameters, such as fatigue, pain, and mobility impairment, predicted both low overall and health-related QoL (26). Regarding chronic conditions, it should be noted that their progression is slow and long-lasting, with an increasing number of individuals with such conditions that may adversely affect one’s health-related QoL (27). However, aside from the fact that chronic disease can adversely affect QoL, particularly in older people, QoL is a complex construct with several dimensions, including physical and mental health, personal perspectives, social and economic factors, and environmental factors (28). Additionally, multimorbidity was reported to have the highest prevalence among older adults with an unfavorable consequence such as disability, functional decline, high health care costs, and poor QoL (29).

Previously, in a study of elderly individuals from India, it was stated that living without a spouse affects QoL (30). Moreover, in another study from Brazil on active elderly, it was also stressed that marital status has an impact on one’s QoL (31). However, in another study, living alone was not associated with lower QoL, whereas loneliness, as a subjective measure, was associated with lower QoL (32). Although living alone is not the same as feeling lonely, it should be noted that older age is often marked by loneliness (32). Therefore, the influence of marital status on older adults’ QoL should be considered a complex dimension, with various factors that can affect the QoL outcome.

Considering ethnic background, a previous study that included Chinese, Malay, and Indian individuals in Singapore found that ethnicity and socioeconomic status were associated with health-related quality of life (33). Furthermore, in another study that was performed on White British, Asian, Black Caribbean, Black African, and Chinese people aged 55 years and above living in England and Scotland, differences in health, income, and social support were found among the ethnic groups (34). These findings could suggest that factors from different ethnic groups can have varying degrees of influence on one’s QoL and its perception.

Regarding the influence of the living environment on individuals’ quality of life, our findings indicated that living in a rural area in Kazakhstan has more favorable effects on older adults’ QoL. It has been stated in the literature that living environment, whether rural or urban, affects income levels, access to services, and the attention received, thereby impacting the QoL of older adults (35). In a cross-sectional study of community-dwelling older adults in Mongolia, lower levels of life satisfaction were associated with living in rural areas (36). However, in the Canadian Longitudinal Study on Aging, higher levels of satisfaction were reported in the rural population than in urban areas (37). Moreover, in a Nigerian study of community-dwelling older adults, it was reported that, in the environmental domain, QoL scores were significantly higher among those living in rural areas than among those living in urban areas (38). At the same time, no differences were found for the physical, psychological, and social domains of QoL (38). These findings imply that the residing area (urban or rural) might have a complex influence on individuals’ QoL; thus, specific and targeted interventions should be proposed and implemented, bearing in mind numerous factors that can influence the overall QoL, particularly in older adults, to improve their wellbeing, satisfaction, and achieve optimal functioning.

Regarding gender during the tested time period in 2022, our findings from univariate linear regression showed that better self-reported overall health and the absence of cardiovascular disease in both genders were significantly associated with better quality of life, whereas chronic diseases and belonging to an ethnic group other than Kazakh in both genders were associated with decreased quality of life. In men, being married was significantly associated with a better quality of life. In women, the absence of hypertension and the absence of chronic heart failure, as well as a rural place of residence, were associated with better quality of life, while the presence of COPD was associated with decreased quality of life. Additionally, we have pointed out that in male participants, aging with diabetes as well as being single significantly worsens the quality of life, while in female participants, aging with COPD, chronic diseases, and belonging to an ethnic background other than Kazakh significantly worsens their quality of life.

It was previously reported that women, compared to men, have more chronic conditions, which, despite causing suffering, threaten life less than heart disease and cancer, which are seen more in men (39). Moreover, it has been stated that, in addition to biological differences between genders, cultural and social structures have a greater influence on gender differences in determining quality of life (39). In our study, we demonstrated that the strongest interaction between age and other variables in both genders was with ethnic background for women, where they had a significantly lower quality of life as they aged compared to their male counterparts. Our results imply that different factors might influence the specific degree of quality of life in men and women; thus, an individualized approach, bearing in mind potential gender differences, should be taken into consideration to adequately address potential factors that can affect the quality of life in both men and women to different degrees.

Regarding the place of residence, univariate linear regression, in subjects from the urban region, better self-reported overall health, absence of hypertension, absence of chronic heart failure, and absence of cardiovascular diseases were significantly associated with better quality of life, while the presence of chronic diseases and belonging to an ethnic group other than Kazakh were significantly associated with decreased quality of life, for those from rural regions, diabetes was significantly associated with decreased quality of life. In the multivariate linear regression model, better self-reported overall health and the absence of chronic heart failure were significantly associated with better quality of life, whereas the presence of chronic diseases and belonging to an ethnic group other than Kazakh were associated with decreased quality of life among participants from urban regions.

For participants from urban areas in our study, aging in combination with chronic heart failure, existing chronic diseases, and belonging to ethnic groups other than Kazakh significantly worsened quality of life. In contrast, for participants from rural areas, aging in combination with hypertension, as well as with chronic diseases, significantly worsened quality of life. These findings suggest that people living in urban areas are exposed to more factors that can affect their quality of life.

In a study of older adults in urban communities in Thailand, good family support was associated with better COVID-19 preventive behaviors (40). Additionally, it was reported that face-to-face communication became less frequent and physical activity decreased more often among rural individuals (41). All of this underscores the complexity of the factors affecting QoL among older people living in urban and rural areas.

### Limitations

The design of the study is cross-sectional, thus limiting causal analysis evaluation. We evaluated the quality of life of elderly patients in 2022 in the context of the COVID-19 pandemic without the impact of the pandemic. Self-reported data are used in this study; therefore, certain biases should be pointed out, including proposed measurement misunderstanding and social-desirability bias (42). Moreover, only participants from one region of Kazakhstan (Almaty city and Almaty region) were included in the sampling process; therefore, potential sampling limitations and generalizability difficulties can exist. Additionally, small sample sizes of some subcategories for certain variables are present, thus potentially limiting statistical power in selective subgroup analysis. Furthermore, additional variables including overall mortality, prolonged hospitalization when needed, and long COVID should also be included in future studies. Moreover, to evaluate the impact of the pandemic on the quality of life in the elderly, additional variables should be evaluated, such as fear of infection, social effects of the closures and quarantine, and the use of masks.

### Policy and practice implications

Healthcare policies must emphasize the prevention and control of chronic illnesses, especially among the elderly. In this case, it includes boosting access to regular screening, low-cost drugs, and patient education programs. Social programs that provide help to reduce loneliness and provide emotional and practical support to single elderly individuals are also essential.

The Republic of Kazakhstan, within the State Program for Healthcare Development 2020–2025, focuses on additional domains, including healthy lifestyle, development of public health services, and improvement of medical care quality and sustainable development of the healthcare system (43, 44).

This study further indicates gender- and place-of-residence-differentiated predictors of QoL. These results call for gender and place-of-residence-responsive healthcare provision and community support services.

Given that ethnic background was significantly associated with quality of life across all interaction models—including age, gender, and place of residence—it can be assumed that special attention should be directed toward better understanding the factors underlying this association. These interaction effects highlight the importance of considering multiple sociodemographic and health-related factors simultaneously, which is a key advantage of the general linear model. In particular, future efforts should consider the roles of health perception, social integration, and socioeconomic resources, as well as propose and implement optimal measures to reduce the impact and burden of these factors.

This finding shows that rural-dwelling older people reported higher QoL than their urban-dwelling counterparts, suggesting the need to study and improve living conditions in urban settings. Urban planning and service delivery need to be better aligned with the needs of aging populations. Healthcare and social services should be more readily available and adaptable to the needs of aging individuals.

Therefore, policymakers and physicians should use collaborative, culturally valid approaches to address the needs of aging people and alleviate declines in quality of life during and well after government health crises, such as the COVID-19 pandemic.

## Conclusion

The findings of our study indicate that quality of life in older adults varies according to the level of self-reported overall health, presence of chronic diseases, marital status, ethnicity, and place of residence. Better quality of life was reported in older adults with better self-reported overall health, those without chronic diseases, as well as those without hypertension, chronic heart failure and cardiovascular disease, older adults who are married, belonging to the Kazakh ethnic group, and living in rural areas.

Subgroup analyses indicated that the influence of health-related and sociodemographic factors differed by gender and place of residence in older adults. For men, marital status and ethnic background were identified as factors associated with the quality of life, whereas among women, COPD and ethnic background were pointed out as factors associated with the quality of life. Differences were also observed between urban and rural residents, with self-reported overall health, presence of chronic diseases, chronic heart failure, and ethnic background being more relevant in urban areas and diabetes in rural areas.

These findings should be interpreted with caution, as they reflect associations observed within the context of the study design. Overall, the results suggest the potential need for preventive and interventional strategies in healthcare and social care settings that account for both gender and place of residence.

## Data Availability

The datasets presented in this article are not readily available because data is available upon reasonable request from the author Dinara Sukenova. Requests to access the datasets should be directed to Dinara Sukenova, sukenova.d@kaznmu.kz.

## References

[1] Johns Hopkins Coronavirus Resource Center. COVID-19 data from around the world countries Kazakstan 2024. Available online at: https://coronavirus.jhu.edu/region/kazakhstan (Accessed March 17, 2025)

[2] Marzo RR Khanal P Ahmad A Rathore FA Chauhan S Singh A . Quality of life of the elderly during the COVID-19 pandemic in Asian countries: a cross-sectional study across six countries. Life (Basel). (2022) 12:365. doi: 10.3390/life12030365, 35330116 PMC8948612

[3] QAZSTAT. Older persons of Kazakhstan. Population and housing census data 2021. Available online at: https://stat.gov.kz/upload/medialibrary/4c8/dz4zth1jqmuclyt2qqbkaqt1x20s9end/POZH0А.pdf (Accessed March 27, 2025)

[4] Khorani H Mohammadi F Hosseinkhani Z Motalebi SA. Predictive factors of quality of life in older adults during the COVID-19 pandemic. BMC Psychol. (2022) 10:176. doi: 10.1186/s40359-022-00882-w, 35843952 PMC9288663

[5] Said CM Batchelor F Duque G. The impact of the COVID-19 pandemic on physical activity, function, and quality of life. Clin Geriatr Med. (2022) 38:519–31. doi: 10.1016/j.cger.2022.04.003, 35868670 PMC9023337

[6] Markotegi M Irazusta J Sanz B Rodriguez-Larrad A. Effect of the COVID-19 pandemic on the physical and psychoaffective health of older adults in a physical exercise program. Exp Gerontol. (2021) 155:111580. doi: 10.1016/j.exger.2021.111580, 34601075 PMC8492068

[7] Siltanen S Ilmarinen K Luoma ML Leppäaho S Kehusmaa S. Changes in older people's quality of life in the COVID-19 era: a population-based study in Finland. Qual Life Res. (2022) 31:3177–87. doi: 10.1007/s11136-022-03210-2, 36057938 PMC9440997

[8] Sürmeli Y Can AA Coşkun G Yılmaz DV. Factors affecting quality of life in elderly people during the COVID-19 pandemic: a cross-sectional study. Mediterr Nurs Midwif. (2024) 4:139–45. doi: 10.4274/MNM.2024.23185

[9] Carmona-González M Flores-Garnica A Sánchez-Ramos MÁ Ortiz-Rodríguez MA Arenas-Ocampo ML García-Serrano LA . Impact of the COVID-19 pandemic on the quality of life of older adults. J Glob Health Rep. (2022) 6:e2022032. doi: 10.29392/001c.37468

[10] Salci MA Carreira L Baccon WC Marques FRDM Höring CF Oliveira MLF . Perceived quality of life and associated factors in long COVID syndrome among older Brazilians: a cross-sectional study. J Clin Nurs. (2024) 33:178–91. doi: 10.1111/jocn.16618, 36680417

[11] Briere J Wang SH Khanam UA Lawson J Goodridge D. Quality of life and well-being during the COVID-19 pandemic: associations with loneliness and social isolation in a cross-sectional, online survey of 2,207 community-dwelling older Canadians. BMC Geriatr. (2023) 23:615. doi: 10.1186/s12877-023-04350-x, 37777717 PMC10542692

[12] Lee KH Xu H Wu B. Gender differences in quality of life among community-dwelling older adults in low- and middle-income countries: results from the study on global AGEing and adult health (SAGE). BMC Public Health. (2020) 20:114. doi: 10.1186/s12889-020-8212-0, 31992264 PMC6988191

[13] Lima MG Barros MB César CL Goldbaum M Carandina L Ciconelli RM. Impact of chronic disease on quality of life among the elderly in the state of São Paulo, Brazil: a population-based study. Rev Panam Salud Publica. (2009) 25:314–21. doi: 10.1590/s1020-49892009000400005, 19531319

[14] Kim J Lee M Dan H. Gender differences in factors affecting life satisfaction of the elderly with multimorbidity in Korea. Nurs Rep. (2021) 11:54–63. doi: 10.3390/nursrep11010006, 34968312 PMC8608087

[15] Violan C Foguet-Boreu Q Flores-Mateo G Salisbury C Blom J Freitag M . Prevalence, determinants and patterns of multimorbidity in primary care: a systematic review of observational studies. PLoS One. (2014) 9:e102149. doi: 10.1371/journal.pone.0102149, 25048354 PMC4105594

[16] Mouratidis K. How COVID-19 reshaped quality of life in cities: a synthesis and implications for urban planning. Land Use Policy. (2021) 111:105772. doi: 10.1016/j.landusepol.2021.105772, 34566233 PMC8456312

[17] Mouratidis K. COVID-19 and the compact city: implications for well-being and sustainable urban planning. Sci Total Environ. (2022) 811:152332. doi: 10.1016/j.scitotenv.2021.152332, 34914991 PMC8666382

[18] Greteman BB Garcia-Auguste CJ Gryzlak BM Kahl AR Lutgendorf SK Chrischilles EA . Rural and urban differences in perceptions, behaviors, and health care disruptions during the COVID-19 pandemic. J Rural Health. (2022) 38:932–44. doi: 10.1111/jrh.12667, 35466479 PMC9115219

[19] Bureau of National statistics. Agency for strategic planning and reforms of the Republic of Kazakhstan. Bureau of National Statistics. Available online at: https://www.stat.gov.kz/ (Accessed March 13, 2021)

[20] International Longevity Center UK. Older people’s quality of life questionnaire (OPQOL-35). Available online at: https://ilcuk.org.uk/wp-content/uploads/2019/03/OPQOL-full-questionnaire.pdf (Accessed March 27, 2025).

[21] Bilotta C Bowling A Nicolini P Casè A Pina G Rossi SV . Older people's quality of life (OPQOL) scores and adverse health outcomes at a one-year follow-up. A prospective cohort study on older outpatients living in the community in Italy. Health Qual Life Outcomes. (2011) 9:72. doi: 10.1186/1477-7525-9-72, 21892954 PMC3175435

[22] Cohen JW. Statistical power analysis for the behavioral sciences. 2nd ed. Hillsdale, NJ: Lawrence Erlbaum Associates (1988).

[23] Akanov AA Tulebaev KA Eshmanova AK Chaĭkovskaia VV Abikulova AK Kalmakhanov SB . Analysis of condition and prospects in geriatric care of population of Kazakhstan. Adv Gerontol. (2014) 27:589–95. 25827013

[24] Asian Development Bank. Regional well-being across Kazakhstan. Harnessing survey data for inclusive development. Asian Development Bank. Mandaluyong City, Philippines, 2023. Available online at: https://www.adb.org/sites/default/files/publication/892586/regional-well-being-kazakhstan-survey-data.pdf (accessed November 05, 2025)

[25] Panzabekova AZ Digel IE. Factors affecting life expectancy in Kazakhstan. R-Economy. (2020) 6:261–70. doi: 10.15826/recon.2020.6.4.023

[26] Borglin G Jakobsson U Edberg AK Hallberg IR. Self-reported health complaints and their prediction of overall and health-related quality of life among elderly people. Int J Nurs Stud. (2005) 42:147–58. doi: 10.1016/j.ijnurstu.2004.06.003, 15680613

[27] Megari K. Quality of life in chronic disease patients. Health Psychol Res. (2013) 1:e27. doi: 10.4081/hpr.2013.e27, 26973912 PMC4768563

[28] Rezq KA Algamdi MM Alatawi F Altamimi D Albalawi N Albalawi A . Predictors for the level of quality of life among older adults: approaches to the effect of sociodemographic and chronic diseases. J Aging Res. (2024) 2024:5436660. doi: 10.1155/jare/5436660, 39620195 PMC11608296

[29] Makovski TT Schmitz S Zeegers MP Stranges S van den Akker M. Multimorbidity and quality of life: systematic literature review and meta-analysis. Ageing Res Rev. (2019) 53:100903. doi: 10.1016/j.arr.2019.04.005, 31048032

[30] Kumar SG Majumdar AGP. Quality of life (QOL) and its associated factors using WHOQOL-BREF among elderly in urban Puducherry, India. J Clin Diagn Res. (2014) 8:54–7. doi: 10.7860/JCDR/2014/6996.3917, 24596723 PMC3939587

[31] Alexandre TS Cordeiro RC Ramos LR. Factors associated to quality of life in active elderly. Rev Saude Publica. (2009) 43:613–21. doi: 10.1590/s0034-89102009005000030 19488665

[32] Lim LL Kua EH. Living alone, loneliness, and psychological well-being of older persons in Singapore. Curr Gerontol Geriatr Res. (2011) 2011:673181. doi: 10.1155/2011/673181, 21969827 PMC3182578

[33] Thumboo J Fong KY Machin D Chan SP Soh CH Leong KH . Quality of life in an urban Asian population: the impact of ethnicity and socio-economic status. Soc Sci Med. (2003) 56:1761–72. doi: 10.1016/s0277-9536(02)00171-5, 12639592

[34] Moriarty J Butt J. Inequalities in quality of life among older people from different ethnic groups. Ageing Soc. (2004) 24:729–53. doi: 10.1017/S0144686X04002521

[35] Rey-Beiro S Martínez-Roget F. Rural-urban differences in older adults' life satisfaction and its determining factors. Heliyon. (2024) 10:e30842. doi: 10.1016/j.heliyon.2024.e30842, 38774093 PMC11107240

[36] Otgon S Burnette D Mukhtar Y Casati F Myagmarjav S. Life satisfaction among older adults in rural and urban Mongolia: a cross-sectional survey study. Biomed Hub. (2023) 8:79–87. doi: 10.1159/000533917, 37900971 PMC10601859

[37] St John PD Menec V Tate R Newall N Cloutier D O'Connell ME. Life satisfaction in adults in rural and urban regions of Canada—the Canadian longitudinal study on aging. Rural Remote Health. (2021) 21:6631. doi: 10.22605/RRH6631, 34454411

[38] Cadmus EO Adebusoye LA Owoaje ET. Rural–urban differences in quality of life and associated factors among community-dwelling older persons in Oyo state, South-Western Nigeria. Qual Quant. (2022) 56:1327–44. doi: 10.1007/s11135-021-01178-8

[39] Carmel S. Health and well-being in late life: gender differences worldwide. Front Med (Lausanne). (2019) 6:218. doi: 10.3389/fmed.2019.00218, 31649931 PMC6795677

[40] Yodmai K Pechrapa K Kittipichai W Charupoonpol P Suksatan W. Factors associated with good COVID-19 preventive behaviors among older adults in urban communities in Thailand. J Prim Care Community Health. (2021) 12:21501327211036251. doi: 10.1177/21501327211036251, 34334008 PMC8326623

[41] Sauliune S Kalediene R Kalibatas V Kaseliene S Mesceriakova O. Lifestyle changes among older adults during and after COVID-19 pandemic in Lithuania. Front Public Health. (2025) 12:1504193. doi: 10.3389/fpubh.2024.1504193, 39835321 PMC11743638

[42] Rosenman R Tennekoon V Hill LG. Measuring bias in self-reported data. Int J Behav Healthc Res. (2011) 2:320–32. doi: 10.1504/IJBHR.2011.043414, 25383095 PMC4224297

[43] State program for the development of health Care of the Republic of Kazakhstan for 2020–2025. Available online at: https://cis-legislation.com/document.fwx?rgn=121946 (accessed November 16, 2025).

[44] Gulis G Aringazina A Sangilbayeva Z Kalel Z de Leeuw E Allegrante JP. Population health status of the Republic of Kazakhstan: trends and implications for public health policy. Int J Environ Res Public Health. (2021) 18:12235. doi: 10.3390/ijerph182212235, 34831990 PMC8621160

